# Supplementary material for: The Impact of Emergency Department Arrival Time on Door-to-Balloon Time in Patients with ST-Segment Elevation Myocardial Infarction Receiving Primary Percutaneous Coronary Intervention
Source: J Clin Med. 2023 Mar 20;12(6):2392. doi: 10.3390/jcm12062392 (PMC10059039; doi:10.3390/jcm12062392)
Supplement: Supplementary file 1 [file jcm-12-02392-s001.zip › jcm-2254251-supplementary.pdf]

Supplementary Table S1. Sensitivity analysis for the predictors associated with different time intervals during door–to–balloon time in ED STEMI patients

	**Door–to–Balloon Time > 90 min**			**Door–to–ECG > 10 min**			**ECG–to–Consultation > 10 min**			**Consultation–to–Activation > 10 min**			**Activation–to–Cath Lab-Arrival > 30 min**			**Cath Lab–Arrival–to–Balloon Time > 30 min**		
**Parameters**	**aOR**	**(95% CI)**	***P* value**	**aOR**	**(95% CI)**	***P* value**	**aOR**	**(95% CI)**	***P* value**	**aOR**	**(95% CI)**	***P* value**	**aOR**	**(95% CI)**	***P* value**	**aOR**	**(95% CI)**	***P* value**
**Age**				1.03	(1.00–1.06)	0.030												
**Female sex**	2.83	(1.56–5.13)	<0.001	3.23	(1.63–6.39)	<0.001												
**Weekend**													2.08	(1.40–3.07)	<0.001			
**Year**																		
2013		Reference						Reference			Reference			Reference			Reference	
2014	1.32	(0.56–3.12)	0.526				0.54	(0.26–1.12)	0.096	3.29	(0.66–16.37)	0.145	2.20	(0.78–6.20)	0.137	1.55	(0.79–3.05)	0.200
2015	0.60	(0.22–1.65)	0.320				0.77	(0.37–1.59)	0.475	4.17	(0.84–20.83)	0.082	2.83	(0.98–8.15)	0.054	1.85	(0.92–3.73)	0.084
2016	0.41	(0.14–1.19)	0.102				0.37	(0.16–0.81)	0.014	4.05	(0.83–19.72)	0.083	3.26	(1.18–8.98)	0.023	0.75	(0.36–1.56)	0.438
2017	0.49	(0.17–1.37)	0.174				0.52	(0.24–1.09)	0.081	6.25	(1.35–28.98)	0.019	2.70	(0.97–7.49)	0.057	0.61	(0.28–1.32)	0.208
2018	0.17	(0.04–0.65)	0.010				0.26	(0.11–0.63)	0.003	2.97	(0.58–15.22)	0.191	3.81	(1.39–10.45)	0.009	0.65	(0.30–1.38)	0.257
2019	0.58	(0.23–1.47)	0.249				0.92	(0.47–1.80)	0.804	6.42	(1.41–29.29)	0.016	4.69	(1.75–12.61)	0.002	0.67	(0.22–1.02)	0.055
2020	0.28	(0.09–0.90)	0.032				0.57	(0.27–1.20)	0.139	8.19	(1.79–37.41)	0.007	10.68	(4.00–28.50)	<0.001	0.37	(0.16–0.88)	0.024
2021	0.42	(0.14–1.30)	0.131				0.83	(0.40–1.72)	0.613	8.411	(1.82–38.83)	0.006	17.36	(6.40–47.07)	<0.001	0.59	(0.27–1.32)	0.201
**Triage level**																		
**1**		Reference			Reference													
**2**	0.72	(0.34–1.56)	0.407	0.44	(0.19–0.99)	0.049												

**3**	3.20	(1.23–8.34)	0.017	3.05	(1.17–7.95)	0.023												
**Lateral wall STEMI**																		
**ED visit time**																		
**08:00–15:59**	Reference			Reference			Reference			Reference			Reference			Reference		
**16:00–23:59**	1.71	(0.92–3.16)	0.088	1.02	(0.51–2.03)	0.956	0.35	(0.21–0.60)	0.477	0.88	(0.51–1.51)	0.639	3.84	(2.44–6.04)	<0.001	0.89	(0.60–1.34)	0.578
**00:00–07:59**	2.95	(1.51–5.78)	0.002	1.38	(0.63–3.01)	0.425	1.17	(0.77–1.77)	0.028	1.45	(0.80–2.61)	0.217	6.45	(3.84–10.82)	<0.001	0.88	(0.53–1.45)	0.613

OR: odds ratio; aOR: adjusted odds ratio; CI: confidence interval; ED: emergency department; STEMI: ST-segment elevation myocardial infarction.
